# Supplementary figures and images for: Newly Developed MAGIC Population Allows Identification of Strong Associations and Candidate Genes for Anthocyanin Pigmentation in Eggplant
Source: Front Plant Sci. 2022 Mar 7;13:847789. doi: 10.3389/fpls.2022.847789 (PMC8940277; doi:10.3389/fpls.2022.847789)

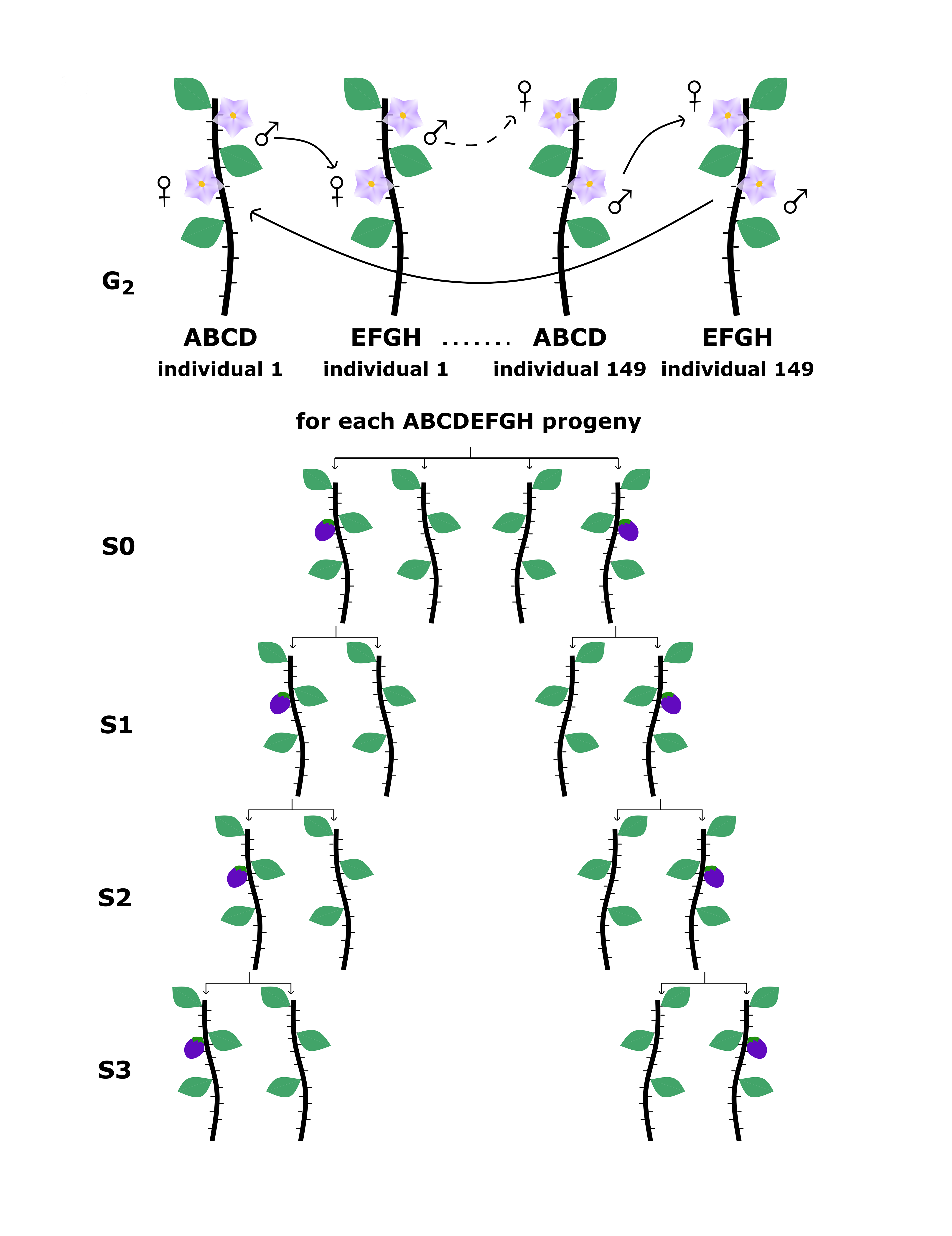

Supplement: Supplementary Figure 1 — (A) Chain pollination scheme of the four-way hybrids followed to obtain the eight-way hybrids. (B) For each S0 progeny, four plants were germinated, selecting for the next generation (S1) only the first two that set fruits with viable seed. For subsequent generations, two plants were germinated and only the first one that set fruit was selected for the next generation. [file Image_1.TIF]

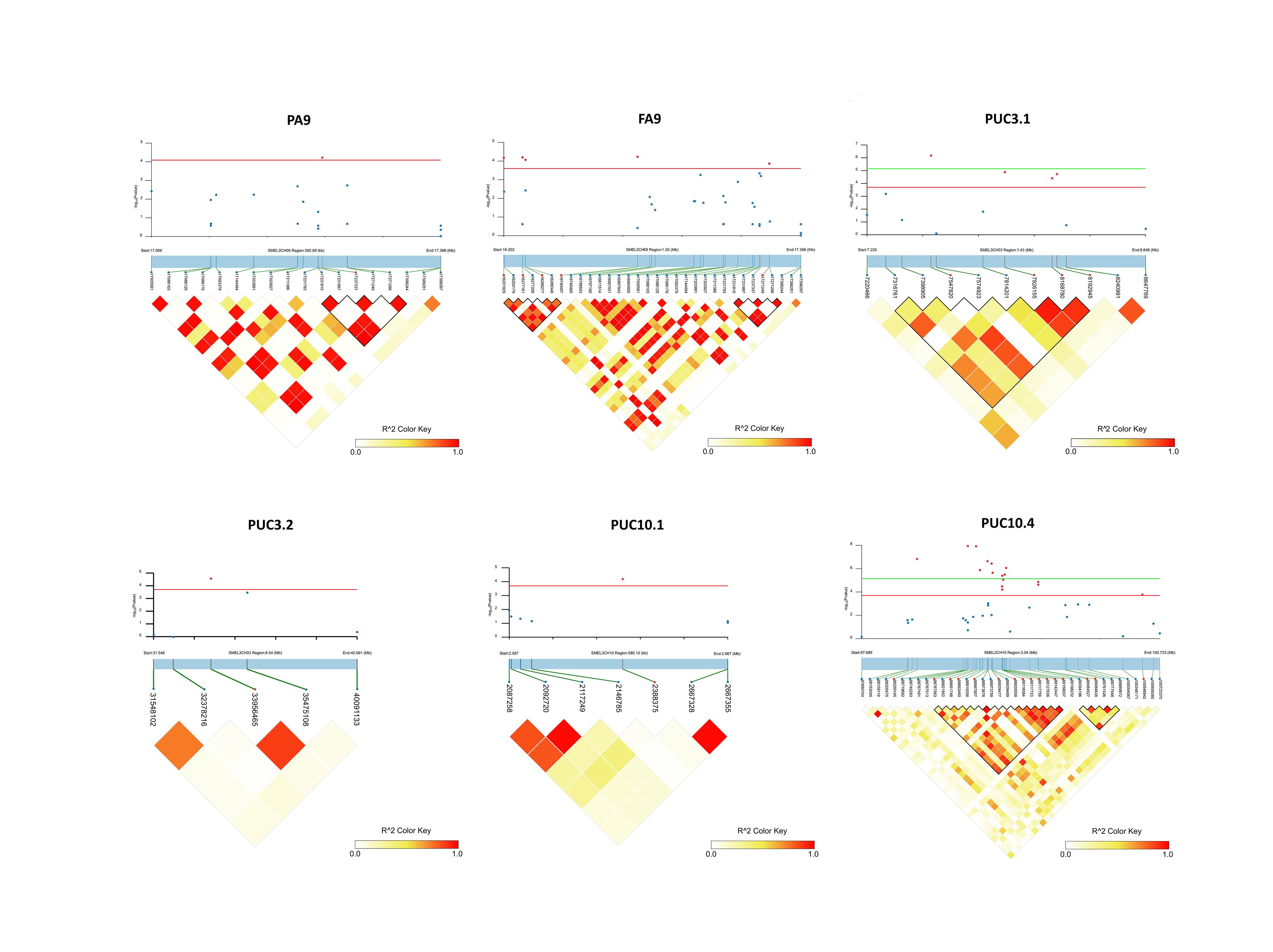

Supplement: Supplementary Figure 2 — Local Manhattan plot (top) and LD heatmap (bottom) surrounding the peaks PA9 (A), FA9 (B), PUC3.1 (C), PUC3.2 (D), PUC10.1 (E), and PUC10.4 (F). The red and green horizontal lines represent, respectively, FDR and Bonferroni significance thresholds. Pairwise LD between SNPs is indicated as values of R2 values: red indicates a value of 1 and white indicates 0. [file Image_2.TIF]

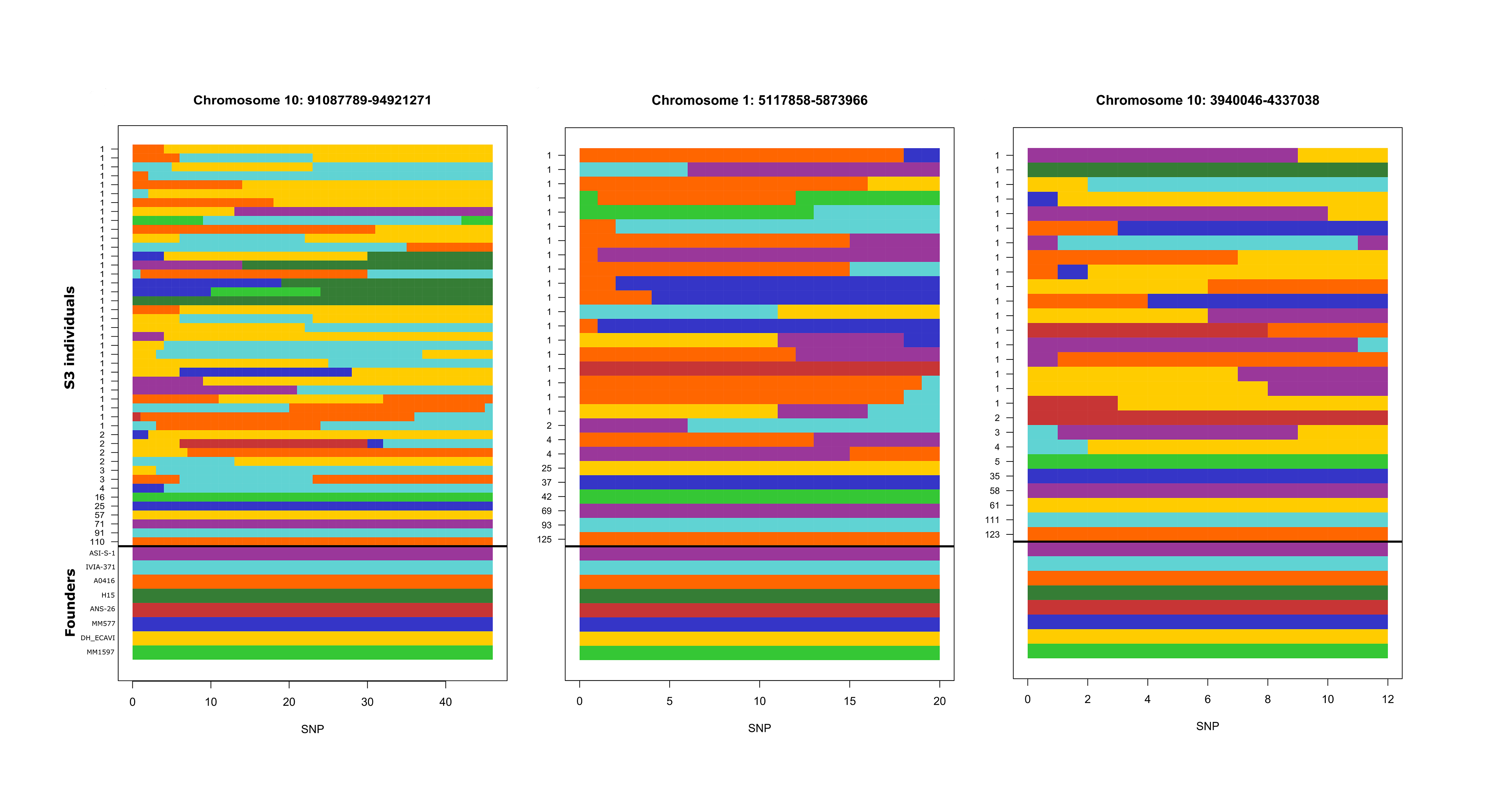

Supplement: Supplementary Figure 3 — Founder haplotype blocks representation predicted for each of the S3 individuals for the three anthocyanin-related candidate gene regions: (A) MYB113 (SMEL_001g120500.1) on chromosome 1 between 5.11 and 5.88 Mb identified by the FA1 and PUC1 associations; (B) COP1 (SMEL_010g339180.1.01) on chromosome 10 between 3.94 and 4.34 Mb identified by the PUC10.2 association; and (C) MYB113 (SMEL_010g351850.1) on chromosome 10 between 91.08 and 94.81 Mb identified by the PA10, FA10, and PUC10.3 associations. [file Image_3.TIF]

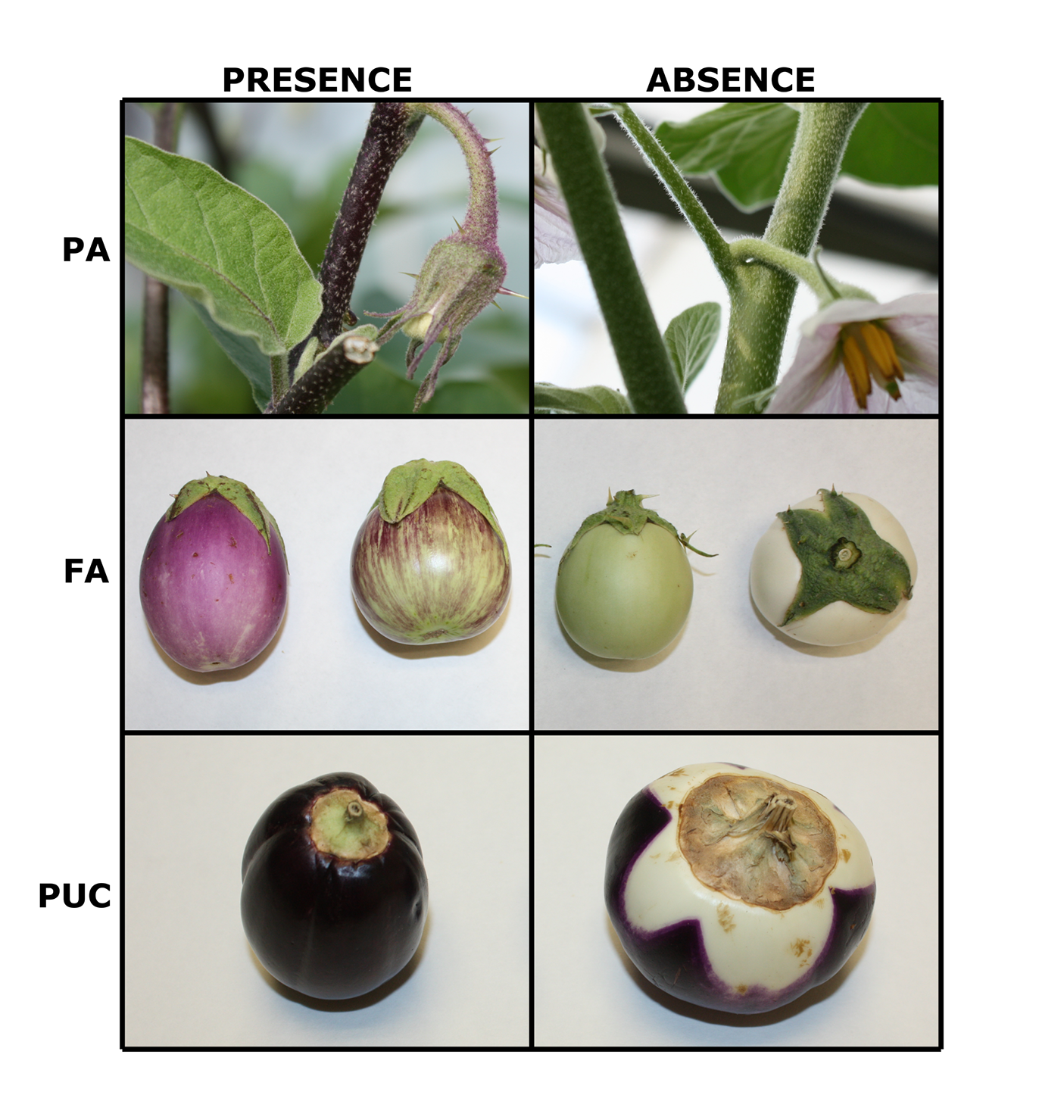

Supplement: Supplementary Figure 4 — Phenotyping of the S3MEGGIC population for presence or absence of PA, FA, and PUC. [file Image_4.TIF]
